# BRG1 Mediates Nephronectin Activation in Hepatocytes to Promote T Lymphocyte Infiltration in ConA-Induced Hepatitis

**DOI:** 10.3389/fcell.2020.587502

**Published:** 2021-01-21

**Authors:** Wenxuan Hong, Ming Kong, Mengwen Qi, Hui Bai, Zhiwen Fan, Ziyu Zhang, Aijun Sun, Xiangshan Fan, Yong Xu

**Affiliations:** ^1^Institute of Biomedical Sciences, Fudan University, Shanghai, China; ^2^Laboratory Center for Experimental Medicine, Department of Clinical Medicine, Jiangsu Health Vocational College, Nanjing, China; ^3^Key Laboratory of Targeted Intervention of Cardiovascular Disease and Collaborative Innovation Center for Cardiovascular Translational Medicine, Department of Pathophysiology, Nanjing Medicine, Nanjing, China; ^4^Department of Pathology, Affiliated Nanjing Drum Tower Hospital of Nanjing University Medical School, Nanjing, China; ^5^Institute of Biomedical Research, Liaocheng University, Liaocheng, China; ^6^Key Laboratory of Women’s Reproductive Health of Jiangxi, Jiangxi Provincial Maternal and Child Health Hospital, Nanchang, China; ^7^Central Laboratory, Jiangxi Provincial Maternal and Child Health Hospital, Nanchang, China

**Keywords:** transcriptional regulation, fulminant hepatitis, T lymphocyte, chemokine, nephronectin

## Abstract

Fulminant hepatitis (FH) is a major cause of acute liver failure. Concanavalin A (ConA) belongs to the lectin family and is frequently used as an inducer of FH in animal models. ConA induced FH is characterized by massive accumulation of T lymphocytes in the liver. A host of chemoattractive substances are known to promote T cell homing to the liver during acute hepatitis. Here we investigated the involvement of Brahma-related gene 1 (BRG1), a chromatin remodeling protein, in FH. BRG1-flox mice were crossed to Alb-Cre mice to generate hepatocyte conditional BRG1 knockout (LKO) mice. The mice were peritoneally injected with a single dose of ConA to induce FH. BRG1 deficiency mitigated ConA-induced FH in mice. Consistently, there were fewer T lymphocyte infiltrates in the LKO livers compared to the wild type (WT) livers paralleling downregulation of T cell specific cytokines. Further analysis revealed that BRG1 deficiency repressed the expression of several chemokines critical for T cell homing including nephronectin (Npnt). BRG1 knockdown blocked the induction of Npnt in hepatocytes and attenuated T lymphocyte migration *in vitro*, which was reversed by the addition of recombinant nephronectin. Mechanistically, BRG1 interacted with β-catenin to directly bind to the *Npnt* promoter and activate *Npnt* transcription. Importantly, a positive correlation between infiltration of CD3^+^ T lymphocyes and nephronectin expression was detected in human acute hepatitis biopsy specimens. In conclusion, our data identify a novel role for BRG1 as a promoter of T lymphocyte trafficking by activating *Npnt* transcription in hepatocytes. Targeting the BRG1-Npnt axis may yield novel therapeutic solutions for FH.

## Introduction

When the liver is challenged with a myriad of pathological stimuli, hepatocytes undergo cell death (apoptosis, necrosis, necroptosis, autophagy, pyroptosis, ferroptosis, etc.) invariably paralleling a compensatory response that aims at containing the damage, regenerating the liver parenchyma, and restoring hepatic normalcy. However, in the event of the most venous attacks that surpass the compensatory/regenerative potential of the liver and cause irreparable damages and permanent loss of liver functions, the host may eventually succumb owing to severe disturbances of homeostasis. Fulminant hepatitis (FH) is one of the major causes for acute liver failure. Defined as a deterioration of liver function and the development of hepatic encephalopathy within 8 weeks after the onset of jaundice, FH can be triggered by viral infection and drug overdose (Ichai and Samuel, 2008). Despite the advances in preventive, diagnostic, and interventional techniques, prognosis for FH patients remains poor and often necessitates liver transplantation, which demands not only better care and management but a clearer understanding of FH pathogenesis (Fujiwara et al., 2015; Ndekwe et al., 2017; Wendon et al., 2017).

Concanavalin A (ConA) belongs to the family of lectins and has been widely used as an inducer of FH in model animals since 1992 (Tiegs et al., 1992). One unique feature regarding ConA-induced FH is that hepatic inflammation in this model is exclusively mediated by recruitment and activation of T lymphocytes (Mizuhara et al., 1994). Depending on the dosing scheme, volcanic inflammatory response can occur in the liver as early as 1 h after ConA injection with massive infiltration of T lymphocytes and concomitant upregulation of T cell specific cytokines (Heymann et al., 2015). Depletion of T lymphocytes, aided by the *Lck*-Cre driven expression of a cytocidal toxin intermedilysin, completely abolishes ConA-induced hepatitis in mice (Feng et al., 2016). Congruently, ablation of interferon gamma (IFN-γ, encoded by IFNG), a prototypical T cell cytokine, or its cognate receptor (IFNGR1/R2), or its downstream mediator IRF-1 attenuates ConA-induced hepatitis in mice (Ohta et al., 2000; Hong et al., 2002; Jaruga et al., 2004). A number of chemokines have been identified to mediate the navigation of T lymphocytes to the liver after the onset of FH including C-C motif ligand 2 (CCL2), macrophage inflammatory protein 1 (MIP1), nephronectin (NPNT), and C-X-C motif ligand 9 (CXCL9) (Leifeld et al., 2003; Saiman and Friedman, 2012; Inagaki et al., 2013; Ikeda et al., 2014). The transcriptional regulation of these cytokines in the context of FH is not completely understood.

Brahma-related gene 1 (BRG1) is a chromatin remodeling protein playing fundamental roles modulating host immunity via immune cell-autonomous and non-autonomous pathways (Smale et al., 2014). For instance, conditional deletion of BRG1 in thymocytes (by the *Lck*-Cre) causes severe abnormalities of T cell maturation and leads to compromised adaptive immunity in mice (Gebuhr et al., 2003). Ablation of BRG1 in mature T lymphocytes (by the *Cd4*-Cre and the *Foxp3*-Cre) is associated with a deficiency in regulatory T cell (Treg) differentiation and renders the mice susceptible to autoimmune disease (Chaiyachati et al., 2013). Alternatively, BRG1 can control immune cell behaviors by contributing to the production and secretion of immunomodulatory molecules in non-immune cells. We have previously investigated the role of BRG1 in immune cell trafficking and found that BRG1-mediated transcription of chemokines and adhesion molecules promotes the mobilization of immune cells in animal models of abdominal aortic aneurysm (Zhang et al., 2018a), atherosclerosis (Fang et al., 2013), cardiac ischemia-reperfusion injury (Zhang et al., 2018b), renal ischemia-reperfusion injury (Liu et al., 2019a), obstructive nephropathy (Liu et al., 2019b), and cardiac hypertrophy (Li et al., 2020c). The role of BRG1 in the pathogenesis of FH, especially in T lymphocyte infiltration, remains to be determined. Here we report that mice with hepatocyte-conditional deletion of BRG1 are partially protected from ConA induced FH. BRG1 regulates T lymphocyte infiltration by activating transcription of the chemokine nephronectin in hepatocytes. Therefore, our data identify a novel role for BRG1 as a promoter of T lymphocyte trafficking.

## Materials and Methods

### Animals

All animal experiments were reviewed and approved by the Ethics Committee on Humane Treatment of Laboratory Animals of Nanjing Medical University and were performed in accordance with the ethical standards laid down in the 1964 Declaration of Helsinki and its later amendments. *Smarca4*-flox mice (Li et al., 2019c) and *Alb*-Cre mice (Li et al., 2019e) were crossed to generate hepatocyte conditional BRG1 knockout (LKO) mice. To induce FH, 8-week male LKO mice and the WT mice were injected via tail vein with ConA (200 mg/kg). The mice were sacrificed 12 h after the injection.

### Histological Staining

Histological analyses were performed essentially as described before. Paraffin sections were stained with H&E (Sigma) according to standard procedures. Parallel sections were stained for CD3. Briefly, the sections were blocked with 10% normal goat serum for 1 h at room temperature and then incubated with anti-CD3 (Abcam, ab16669, 1:200) antibodies. Staining was visualized by incubation with anti-rabbit secondary antibody (Proteintch, 1:1000) and developed with a streptavidin-horseradish peroxidase kit (Pierce) for 20 min. Pictures were taken using an Olympus IX-70 microscope. Quantifications were performed with Image J by two independent pathologists in a blinded fashion.

### Cell Culture, Plasmids, and Transient Transfection

Human hepatoma cells (HepG2) were maintained in DMEM supplemented with 10% fetal bovine serum (FBS, Hyclone). Human T-cell leukemia cells (Jurkat) were maintained in RPMI-1640 supplemented with 10% FBS. Primary hepatocytes were isolated and cultured as previously described (Fan et al., 2020). Mouse *Npnt* promoter-luciferase constructs (Lanthier et al., 2011) and BRG1 expression constructs have been previously described (Chen et al., 2020c; Li et al., 2020a,b, c). Small interfering RNAs were purchased from Dharmacon. Transient transfections were performed with Lipofectamine 2000. Luciferase activities were assayed 24–48 h after transfection using a luciferase reporter assay system (Promega) as previously described (Wu et al., 2020; Yang et al., 2020a,b). For conditioned media (CM) collection, the cells were switched to and incubated with serum-free media overnight. The next day, the media were collected, centrifuged at 4000 × *g* for 30 min at 4°C using 3-kDa MW cut-off filter units (Millipore) and sterilized through a 0.4-μm filter.

### Protein Extraction and Western Blot

Whole cell lysates were obtained by re-suspending cell pellets in RIPA buffer (50 mM Tris pH7.4, 150 mM NaCl, 1% Triton X-100) with freshly added protease inhibitor (Roche) as previously described (Lv et al., 2020; Mao et al., 2020a,b; Sun et al., 2020). Western blot analyses were performed with anti-BRG1 (Santa Cruz, sc-10768), anti-Npnt (Thermo Fisher, PA5-65600), and anti-β-actin (Sigma, A2228) antibodies. For densitometrical quantification, densities of target proteins were normalized to those of β-actin. Data are expressed as relative protein levels compared to the control group which is arbitrarily set as 1.

### RNA Isolation and Real-Time PCR

RNA was extracted with the RNeasy RNA isolation kit (Qiagen). Reverse transcriptase reactions were performed using a SuperScript First-strand Synthesis System (Invitrogen) as previously described (Zhao et al., 2019; Chen et al., 2020a,b; Dong et al., 2020). Real-time PCR reactions were performed on an ABI Prism 7500 system with the following primers: human *NPNT*, 5′-TGGGGACAGTGCCAACCTTTCT-3′ and 5′-TGTGCTTACAGGGCCGAGGCT-3′; mouse *Npnt*, 5′-GCG GATGAGGAAGTAAAGGAC-3′ and 5′-CCTTTGAAGATGA CGCTTTTG-3′; mouse *Ccl2*, 5′-CCCAATGAGTAGGCTGGA GA-3′ and 5′-AAAATGGATCCACACCTTGC-3′; mouse *Mip1*, 5′-CACCCTCTGTCACCTGCTCAA-3′ and 5′-ATGGCGCTG AGAAGACTTGGT-3′; mouse *Cxcl9*, 5′-TGTGGAGTTCGAG GAACCCT-3′ and 5′-TGCCTTGGCTGGTGCTG-3′; mouse *Cxcl10*, 5′-TCCAGTTAAGGAGCCCTTTTAGACC-3′ and 5′-TGAAATCATCCCTGCGAGCCTAT-3′; mouse *Cxcl12*, 5′-GT CTAAGCAGCGATGGGTTC-3′ and 5′-GAATAAGAAAGCAC ACGCTGC-3′; human *BRG1*, 5′-TCATGTTGGCGAGCTAT TTCC-3′ and 5′-GGTTCCGAAGTCTCAACGATG-3′. Ct values of target genes were normalized to the Ct values of housekeeping control gene (18s, 5′-CGCGGTTCTATTTTGTTGGT-3′ and 5′-TCGTCTTCGAAACTCCGACT-3′ for both human and mouse genes) using the ΔΔCt method and expressed as relative mRNA expression levels compared to the control group (WT mice injected with ConA) which is arbitrarily set as 1.

### Chromatin Immunoprecipitation (ChIP)

Chromatin immunoprecipitation (ChIP) assays were performed essentially as described before (Fan et al., 2019; Kong et al., 2019a,b; Li et al., 2019a, b, c, d, e, f; Liu et al., 2019a; Lu et al., 2019; Shao et al., 2019; Weng et al., 2019; Yang et al., 2019a,b). In brief, chromatin in control and treated cells were cross-linked with 1% formaldehyde. Cells were incubated in lysis buffer (150 mM NaCl, 25 mM Tris pH 7.5, 1% Triton X-100, 0.1% SDS, 0.5% deoxycholate) supplemented with protease inhibitor tablet and PMSF. DNA was fragmented into ∼200 bp pieces using a Branson 250 sonicator. Aliquots of lysates containing 200 μg of protein were used for each immunoprecipitation reaction with anti-BRG1 (Santa Cruz, sc-10768), or pre-immune IgG. For re-ChIP, immune complexes were eluted with the elution buffer (1% SDS, 100 mM NaCO_3_), diluted with the re-ChIP buffer (1% Triton X-100, 2 mM EDTA, 150 mM NaCl, 20 mM Tris pH 8.1), and subject to immunoprecipitation with a second antibody of interest.

### T Lymphocyte Migration Assay

T lymphocyte migration was measured using the Boyden chamber inserts (5 μm, Corning) as previously described (Ottoson et al., 2001). Briefly, Jurkat cells were added to the upper chamber whereas the CM collected from hepatocytes were added to the lower chamber. In certain experiments, recombinant human nephronectin (20 ng/ml, R&D) was directly added to the CM. Migrated T cells were counted in at least five different fields for each well. All experiments were performed in triplicates and repeated three times.

### Enzyme-Linked Immunosorbent Assay

Secreted nephronectin levels were examined by ELISA as previously described (Fan et al., 2019; Li et al., 2019d) using commercially available kits according to vendor’s recommendations (for human nephronectin, CSB-EL016019HU, CUSABIO, and for murine nephronectin, ELM-NPNT-1, Raybiotech).

### Human Acute Hepatitis Specimens

Liver biopsies were collected from patients with acute hepatitis referring to Nanjing Drum Tower Hospital under informed consent. Basic information for the patients is listed in Supplementary Table I. All procedures that involved human samples were approved by the Ethics Committee of Nanjing Drum Tower Hospital and adhered to the principles outlined in the Declaration of Helsinki. The paraffin embedded sections were stained with anti-Npnt (Abcam, ab272549, 1:100) and anti-CD3 (Abcam, ab16669, 1:200). Stainings were scored by two pathologists in a blinded fashion. Patient information is summarized in the Supplementary Table I.

### Statistical Analysis

One-way ANOVA with *post hoc* Scheff’e analyses were performed by SPSS software (IBM SPSS v18.0, Chicago, IL, United States). Unless otherwise specified, values of *p* < 0.05 were considered statistically significant.

## Results

### Hepatocyte BRG1 Deletion Attenuates ConA Induced Hepatitis

**FIGURE 1:**
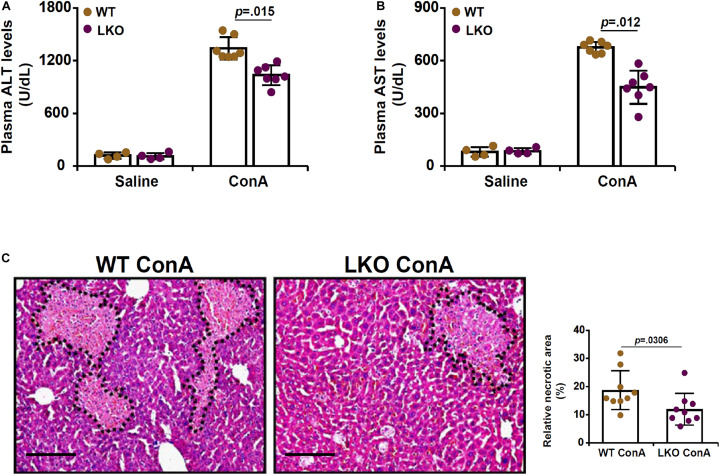
Hepatocyte BRG1 deletion attenuates ConA induced hepatitis. Hepatocyte conditional BRG1 knockout mice (LKO) and wild type (WT) littermates were injected with ConA to induce fulminant hepatitis. The mice were sacrificed at 12 h after the injection. **(A)** Plasma ALT levels. **(B)** Plasma AST levels. **(C)** H&E staining. Dotted lines demarcate necrotic regions. Quantification was performed with Image Pro. *N* = 4–7 mice for each group. Scale bar, 100 μm. Data represent mean ± SD. Student’s *t*-test was used for statistical analyses.

To investigate the role of BRG1 in the pathogenesis of FH, a classic animal model was exploited in which hepatocyte conditional BRG1 knockout (LKO) mice and wild type (WT) littermates were injected with ConA (200 mg/kg) via tail vein (Okamoto et al., 2001). 12 h after the injection, the mice were sacrificed to evaluate liver injury. Measurements of plasma ALT levels (Figure 1A) and AST levels (Figure 1B) showed that ConA injection resulted in drastic elevation of plasma transaminases compared to saline injection. The upregulation of plasma ALT and AST levels was tempered in the LKO mice compared to the WT mice. H&E staining confirmed that there was massive hepatic necrosis in the ConA-injected mice; the LKO mice exhibited smaller necrotic areas than the WT mice (Figure 1C).

### Hepatocyte BRG1 Deletion Attenuates T Lymphocyte Homing to the Liver

**FIGURE 2:**
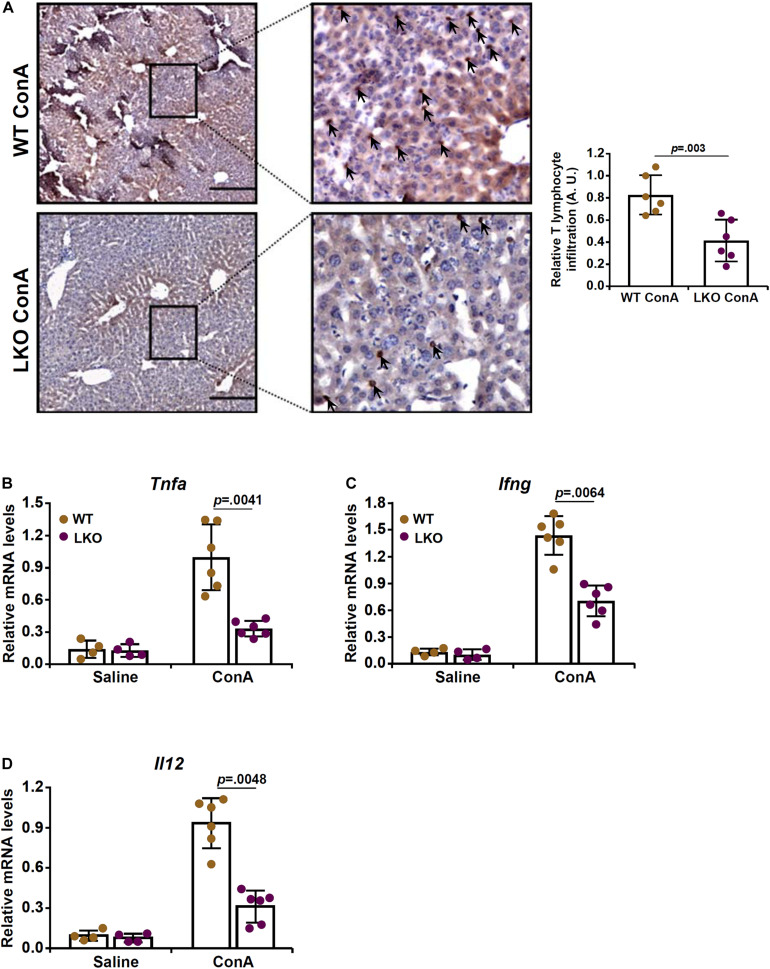
Hepatocyte BRG1 deletion attenuates T lymphocyte homing to the liver. Hepatocyte conditional BRG1 knockout mice (LKO) and wild type (WT) littermates were injected with ConA to induce fulminant hepatitis. The mice were sacrificed at 12 h after the injection. **(A)** Immunofluoresence staining of paraffin sections with an anti-CD3 antibody. **(B–D)** Expression levels of T-cell specific cytokines were examined by qPCR. *N* = 4–7 mice for each group. Scale bar, 100 μm. Data represent mean ± SD. Student’s *t*-test was used for statistical analyses.

Because infiltration of T lymphocytes and the ensuing inflammatory storm play a key role in the pathogenesis of ConA induced FH, we evaluated the effect of hepatocyte-specific BRG1 deletion on T cell homing. Immunohistochemical staining with an anti-CD3 antibody showed that there was massive T cell infiltration in the liver following ConA injection. However, fewer CD3^+^ cells were present in the LKO livers than the WT livers (Figure 2A). Quantitative PCR analysis of T cell signature cytokines revealed that BRG1 deficiency dampened the expression of tumor necrosis factor (*Tnfa*, Figure 2B), IFN-γ (*Ifng*, Figure 2C), and interleukin 12 (*Il12*, Figure 2D) in the liver.

### Hepatocyte BRG1 Deletion Downregulates the Expression of Chemokines Critical for T Lymphocyte Homing

**FIGURE 3:**
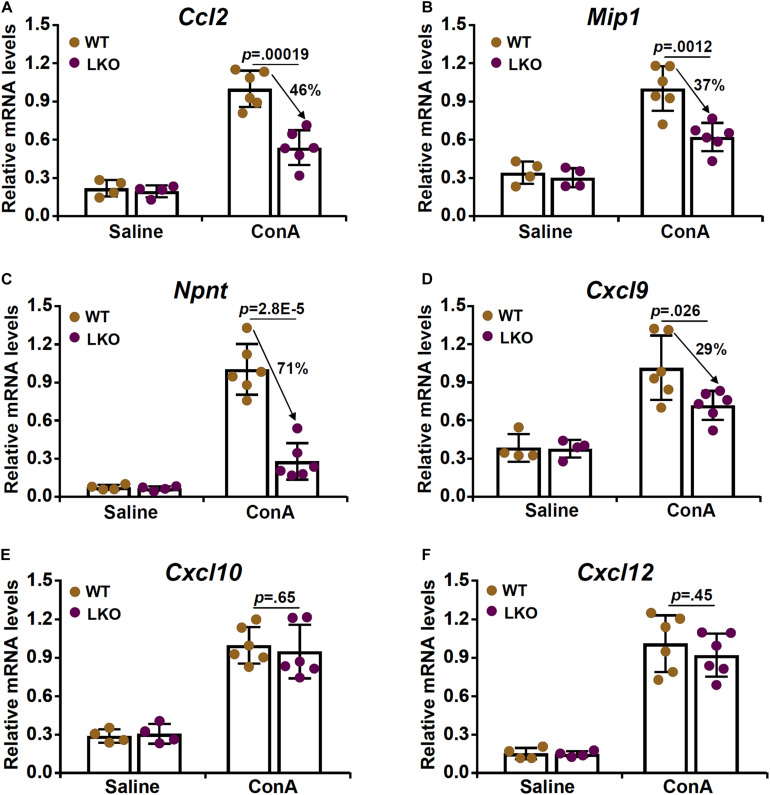
Hepatocyte BRG1 deletion downregulates the expression of chemokines critical for T lymphocyte homing. **(A–F)** Hepatocyte conditional BRG1 knockout mice (LKO) and wild type (WT) littermates were injected with ConA to induce fulminant hepatitis. The mice were sacrificed at 12 h after the injection. Expression levels of Ccl2 **(A)**, Mip1 **(B)**, Npnt **(C)**, Cxcl9 **(D)**, Cxcl10 **(E)**, and Cxcl12 **(F)** were examined by qPCR. *N* = 4–7 mice for each group. Arrows indicate the changes (%) in chemokine expression levels between the WT group and the LKO group. Data represent mean ± SD. Student’s *t*-test was used for statistical analyses.

Based on the observation that BRG1 deletion in hepatocytes attenuated ConA induced acute liver injury likely by suppressing T cell recruitment, we hypothesized that BRG1 might regulate the expression of chemokines critical for T cell chemotaxis. We profiled the expression of several well-known T cell specific chemokines including C-C motif ligand 2 (*Ccl2*, Figure 3A), macrophage inflammatory protein 1 (*Mip1*, Figure 3B), nephronectin (*Npnt*, Figure 3C), C-X-C ligand 9 (*Cxcl9*, Figure 3D), C-X-C ligand 10 (*Cxcl10*, Figure 3E), and C-X-C ligand 12 (*Cxcl12*, Figure 3F). Whereas all six chemokines were upregulated in the liver following ConA injection, BRG1 deletion suppressed the expression of *Ccl2*, *Mip1*, *Npnt*, and *Cxcl9* but not that of either *Cxcl10* or *Cxcl12*. Among the four chemokines whose expression levels were altered by BRG1 deficiency, neprhonectin was the most sensitive to the loss of BRG1 in hepatocytes. Therefore, we focused the regulation of nephronectin by BRG1 for the remainder of the study.

### BRG1 Regulates Nephronectin Expression in Hepatocytes to Promote T Cell Migration

**FIGURE 4:**
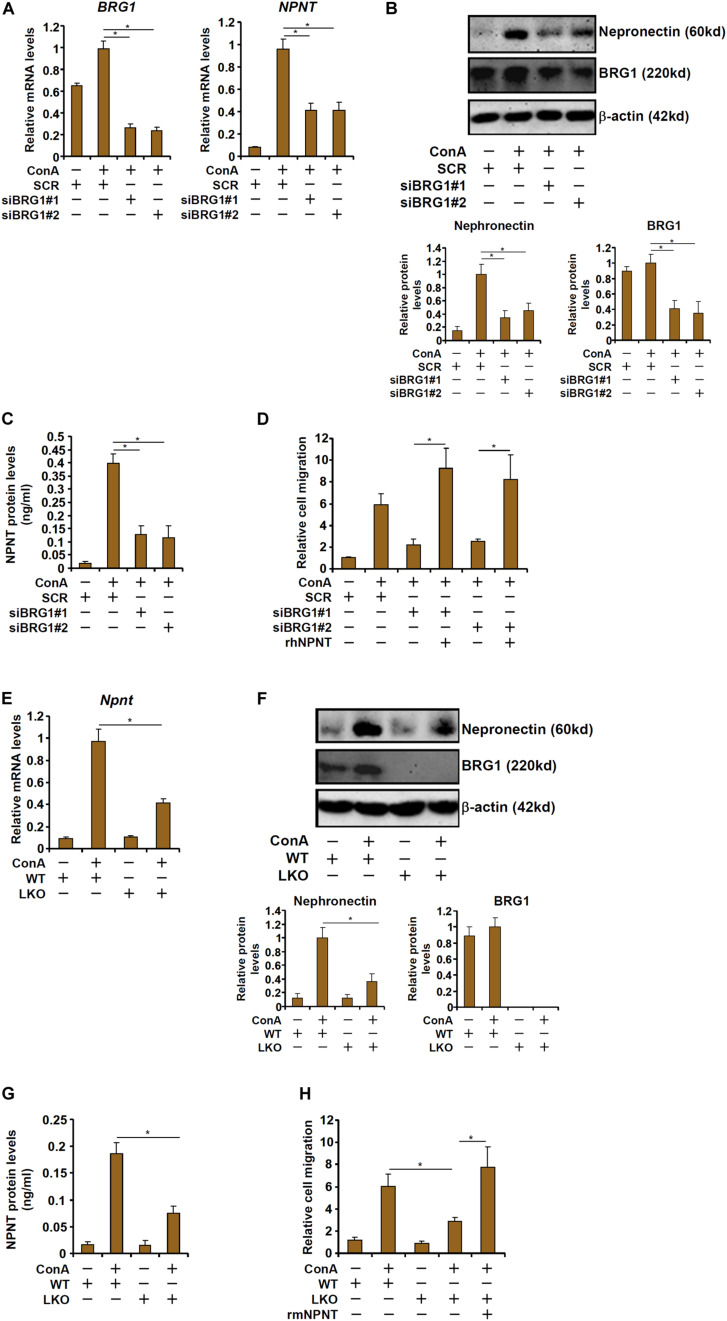
BRG1 regulates nephronectin expression in hepatocytes to promote T cell migration. **(A–D)** HepG2 cells were transfected with siRNA targeting BRG1 or scrambled siRNA (SCR) followed by treatment with ConA. Nephronectin expression was examined by qPCR, Western blotting, and ELISA. T cell migration was evaluated by transwell assay. **(E–H)** Primary hepatocytes were isolated from BRG1 LKO mice and WT mice and treated with ConA. Nephronectin expression was examined by qPCR, Western blotting, and ELISA. T cell migration was evaluated by transwell assay. Data represent averages of three independent experiments and error bars represent SEM. Student’s *t*-test was used for statistical analyses. *p < 0.05.

We then determined whether BRG1 might be essential for ConA induced nephronectin expression in hepatocytes. When HepG2 cells were exposed to ConA, nephronectin expression levels were upregulated by more than 10-fold as measured by qPCR (Figure 4A), Western blotting (Figure 4B), and ELISA (Figure 4C); BRG1 knockdown by two different pairs of siRNAs, however, decreased nephronectin expression by more than 50%. Consistently, CM collected from ConA-treated HepG2 cells robustly enhanced T cell (Jurkat) migration in transwell assay (Figure 4D). BRG1 depletion in HepG2 cells attenuated the chemoattractibility of the CM whereas the addition of exogenous recombinant nephronectin more than compensated for the loss of BRG1 and restored the T cell migration (Figure 4D). In a series of similar experiments, it was observed that ConA treatment induced the expression of nephronectin more potently in primary hepatocytes isolated from WT mice than in those from BRG1 LKO mice as measured by qPCR (Figure 4E), Western blotting (Figure 4F), and ELISA (Figure 4G). Additionally, CM collected from ConA-treated BRG1 LKO hepatocytes stimulated T cell migration less strongly than WT hepatocytes, which could be rescued by the addition of exogenous recombinant nephronectin (Figure 4H). Taken together, these data suggest that BRG1-mediated nephronectin induction by ConA in hepatocytes could contribute to T cell homing.

### BRG1 Directly Activates Nephronectin Transcription in Hepatocytes

**FIGURE 5:**
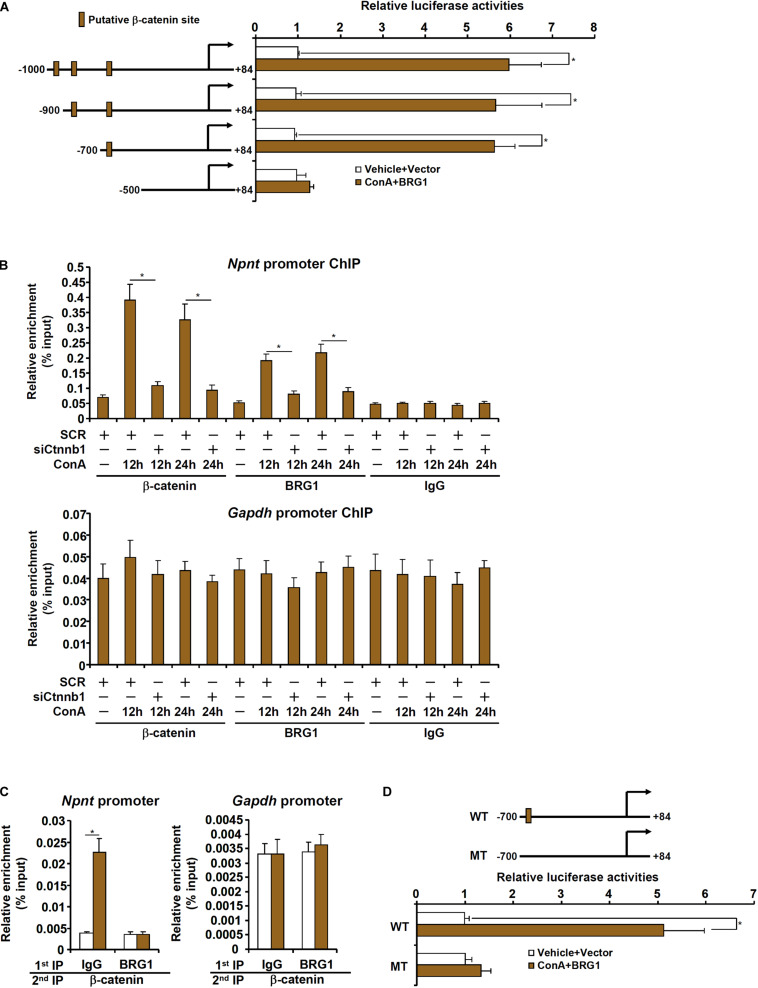
*BRG1 directly activates nephronectin transcription in hepatocytes*. **(A)** HepG2 cells were transfected with *Npnt* promoter-luciferase constructs with or without BRG1 followed by treatment with ConA (20 μg/ml). Luciferase activities were normalized by protein concentration and GFP fluorescence. **(B)** Primary murine hepatocytes were transfected with siRNA targeting β-catenin or scrambled siRNA (SCR) followed by treatment with ConA (20 μg/ml). ChIP assays were performed with anti-β-catenin, anti-BRG1, or IgG. **(C)** Primary murine hepatocytes were treated with or without ConA (20 μg/ml) for 24 h. Re-ChIP assay was performed with indicated antibodies. **(D)** HepG2 cells were transfected with wild type or mutated *Npnt* promoter-luciferase construct with or without BRG1 followed by treatment with ConA (20 μg/ml). Luciferase activities were normalized by protein concentration and GFP fluorescence. Data represent averages of three independent experiments and error bars represent SEM. Student’s *t*-test was used for statistical analyses. *p < 0.05.

We next evaluated the possibility whether BRG1 could directly activate nephronectin transcription. When a mouse *Npnt* promoter-luciferase construct (-1000/+54) was transfected into HepG2 cells, BRG1 over-expression and treatment with ConA robustly activated the promoter activity (Figure 5A). The *Npnt* promoter fragment (-1000/+54) contained a string of binding motifs for β-catenin [Wnt response element (WRE)]. Because we have previously demonstrated that BRG1 interacts with β-catenin to promote proliferation of hepatocytes and liver regeneration, we hypothesized that the same complex might be responsible for the activation of nephronectin transcription by ConA in hepatocytes. A series of inward deletions were introduced into the *Npnt* promoter-luciferase construct to make it progressively shorter and the shortest construct (-500/+54) lost the responsiveness to the induction by BRG1 over-expression plus treatment with ConA (Figure 5A). ChIP assay showed that ConA treatment enhanced the binding of β-catenin and BRG1 on the putative WRE of the *Npnt* promoter but not the *Gapdh* promoter; knockdown of β-catenin simultaneously abrogated the binding of β-catenin and BRG1 (Figure 5B). In addition, mutagenesis of the putative WRE rendered the *Npnt* promoter irresponsive to ConA treatment (Figure 5C). Finally, ConA treatment promoted the formation of a β-catenin-BRG1 complex on the *Npnt* promoter, but not the *Gapdh* promoter, as detected by Re-ChIP assay (Figure 5D). Collectively, these data argue that BRG1 could directly activate *Npnt* transcription likely by interacting with β-catenin.

### Correlation of T Lymphocyte Infiltration and Nephronectin in Human Fulminant Hepatitis Biopsy Specimens

**FIGURE 6:**
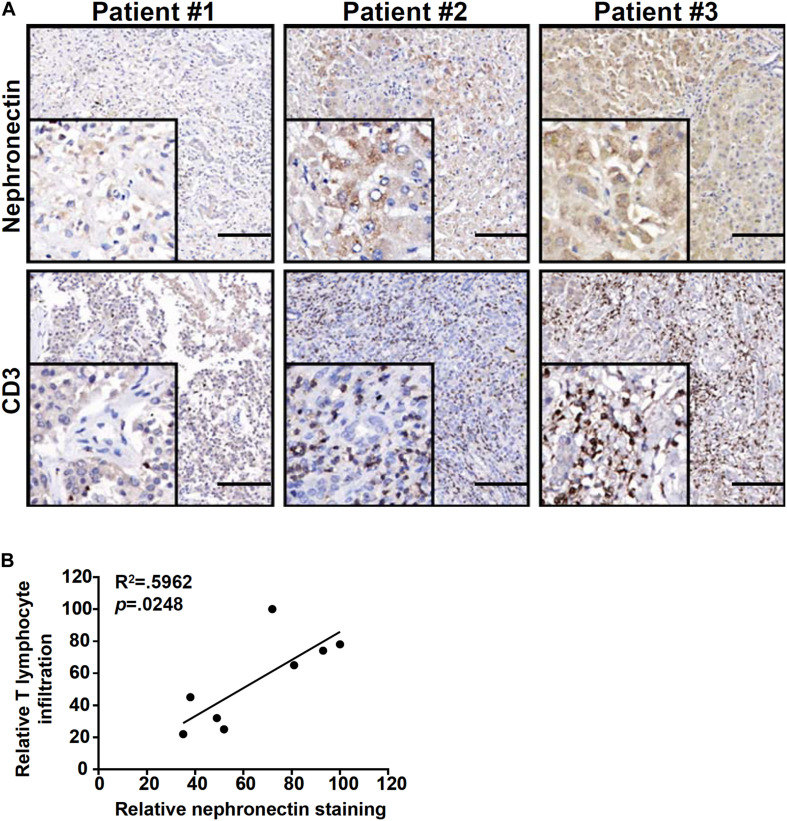
Correlation of T lymphocyte infiltration and nephronectin expression in human fulminant hepatitis biopsy specimens. **(A)** Representative images of CD3 and NPNT staining in liver biopsy specimens of patients diagnosed with fulminant hepatitis. Scale bar, 100 μm. **(B)** Linear regression was performed with Graphpad Prism. Chi-square test was used for statistical analyses.

Finally, we verified the correlation between nephronection expression and T lymphocyte infiltration in biopsy specimens of patients diagnosed with FH. As shown in Figure 6A, more CD3^+^ T lymphocytes were present in the liver where nephronection expression was stronger in a small set of eight specimens examined by immunohistochemical staining. Linear regression determined that there was a significant (*p* = 0.0248) positive correlation between the number of infiltrated T lymphocytes and the magnitude of nephronectin expression (Figure 6B).

## Discussion

T lymphocyte infiltration and the ensuing inflammation play a key role in FH. Here we identify a novel transcriptional pathway that links BRG1-mediated activation of nephronectin to T lymphocyte trafficking in the pathogenesis of FH. BRG1 is known to program the inflammatory response in hepatocytes in various settings. Our group has previously reported that BRG1, recruited by NF-κB, directly binds to the promoters of pro-inflammatory mediators in hepatocytes exposed to excessive nutrient influx and drives hepatic inflammation in the pathogenesis of non-alcoholic steatohepatitis (Tian et al., 2013). Sun et al. (2016) have found that BRG1, as a component of a large chromatin remodeling complex that also including ARID1 and BRM, contributes to liver regeneration at least in part by regulating the transcription of inflammation-related genes in hepatocytes. More recently, Huang et al. (2019) have reported that the non-coding RNA MALAT1 binds to and directs BRG1 to the *IL-6* and *CXCL8* loci in hepatocellular carcinoma (HCC) cells in response to LPS stimulation thereby promoting oncogenic transformation. Combined with our new finding, these observations collectively portray BRG1 as a central integrator of pro-inflammatory signaling pathways in the liver. To date, a genomewide profiling of BRG1-dependent transcription in hepatocytes has yet to be performed. Future studies exploiting transcriptomic techniques including RNA-seq, ChIP-seq, and ATAC-seq would shed additional light on the role of BRG1 in inflammation-associated liver diseases.

Our data demonstrate that β-catenin recruits BRG1 to activate nephronectin (*NPNT*) transcription in hepatocytes. This observation is consistent with the report by Ikehata et al. (2017) that exposure of osteoblasts to the Wnt3a ligand upregulates *NPNT* expression although these authors did not establish *NPNT* as a direct transcriptional target of β-catenin. The pathological relevance of β-catenin in T lymphocytes infiltration during FH remains uncertain. It has been previously shown by Anson et al. (2012) that ConA induced hepatic inflammation is aggravated in the APC-null mice presumably due to stabilization of β-catenin but it was not determined whether nephronectin levels were altered in this model. Neither is it clear whether direct manipulation of β-catenin expression and/or activity would influence liver injury by altering T lymphocyte infiltration in the ConA model. It should be noted that liver-specific deletion of β-catenin appears to render the mice more susceptible to the development of steatosis (Behari et al., 2010) and HCC (Wang et al., 2011), suggesting that any benefit of targeting β-catenin in the intervention of FH has to be weighed against its potential risks. We and others have previously shown that β-catenin relies on BRG1 to regulate the transcription of pro-proliferative genes in hepatocytes and to promote liver regeneration (Li et al., 2019a; Wang et al., 2019). Of note, multiple independent studies have implicated nephronectin in the regulation of cellular proliferation. For instance, Steigedal et al. (2018) have reported that nephronectin promotes proliferation of breast cancer cells by activating the p38-MAPK signaling pathway via its integrin-binding domain (Toraskar et al., 2018). Arai et al. (2017) propose that nephronectin regulates proliferation of dental epithelial stem cells by activating SOX2 expression via the EGF-like repeat domains. In light of our finding as summarized here, the β-catenin-BRG1-nephronectin axis in hepatocytes may possess a broader pathobiological role than mediating T lymphocyte homing during FH and deserves further investigation.

Although we propose that BRG1 activated *NPNT* transcription contributes to T lymphocyte infiltration, there are several lingering questions that await answers. First, in addition to *NPNT*, several other chemokines appear be to downregulated by BRG1 deficiency in the liver (Figure 3). These chemokines might also, at least in part, participate in the recruitment of T lymphocytes. Second, although we show that BRG1 can directly bind to the *NPNT* promoter, it is not clear at this point how BRG1 activates *NPNT* transcription. One of the key mechanisms through which BRG1 regulates transcription is via its extensive interactions with other epigenetic factors including histone and DNA modifying enzymes (Trotter and Archer, 2008). Nephronectin expression is known to be influenced by DNA methylation (Yu et al., 2012), histone acetylation (Wong et al., 2018), and non-coding regulatory RNAs (Kahai et al., 2009; Muller-Deile et al., 2017). It is worthwhile to further examine whether BRG1 could direct the assembly of an epigenetic complex to activate *NPNT* transcription. Third, whereas a correlation between nephronectin expression and T lymphocyte recruitment has been established by the present study, a causative relationship remains to be ascertained. Systemic deletion of nephronectin in mice (*Npnt*^–/–^) results in renal agenesis whereas conditional deletion of nephronectin in nephron progenitor cells in mice (*Npnt*^*f/f*^; *Six2*-Cre) leads to mesangial sclerosis. These animal models should be harnessed to investigate T lymphocyte infiltration in the context of FH.

In summary, our data demonstrate that transcriptional activation of nephronectin by BRG1 may represent a key step in recruitment of T lymphocytes in FH. For the patients with severe FH, effective interventional strategies are still lacking and in many cases require liver transplantation, which poses significant challenge to patient management and care. Because of the prominent role of T lymphocytes in the development and progression of FH, manipulation of T lymphocyte function could potentially yield viable solutions. BRG1 has been known to influence multiple aspects of T lymphocyte function including differentiation, cytokine production, and cytotoxicity (Gebuhr et al., 2003). Our data simply reinforce the notion that BRG1 is a master regulator of T lymphocyte behaviors. Small inhibitors of BRG1 (e.g., PFI-3) have been used in the pre-clinical studies of malignant cancers (Vangamudi et al., 2015). Targeting the newly identified β-catenin-BRG1-nephronectin axis can be considered as a reasonable interventional strategy to treat acute liver injury.

## Data Availability Statement

The original contributions presented in the study are included in the article/Supplementary Material. Further inquiries can be directed to the corresponding author/s.

## Ethics Statement

The studies involving human participants were reviewed and approved by the Nanjing Drum Tower Hospital Ethics Committee on Human Studies. The patients/participants provided their written informed consent to participate in this study. The animal study was reviewed and approved by the Ethics Committee on Humane Treatment of Laboratory Animals of Nanjing Medical University.

## Author Contributions

ZZ, AS, and XF conceived the project. WH, MK, MQ, HB, and ZF designed the experiments, performed the experiments, collected the data, and analyzed the data. YX wrote the manuscript. ZZ, AS, and XF handled funding and supervision. All authors contributed to the article and approved the submitted version.

## Conflict of Interest

The authors declare that the research was conducted in the absence of any commercial or financial relationships that could be construed as a potential conflict of interest.

## References

[B1] Anson M. Crain-Denoyelle A. M. Baud V. Chereau F. Gougelet A. Terris B. (2012). Oncogenic beta-catenin triggers an inflammatory response that determines the aggressiveness of hepatocellular carcinoma in mice. *J. Clin. Invest.* 122 586–599. 10.1172/JCI43937 22251704 PMC3266772

[B2] Arai C. Yoshizaki K. Miyazaki K. Saito K. Yamada A. Han X. (2017). Nephronectin plays critical roles in Sox2 expression and proliferation in dental epithelial stem cells via EGF-like repeat domains. *Sci. Rep.* 7:45181. 10.1038/srep45181 28345658 PMC5366923

[B3] Behari J. Yeh T. H. Krauland L. Otruba W. Cieply B. Hauth B. (2010). Liver-specific beta-catenin knockout mice exhibit defective bile acid and cholesterol homeostasis and increased susceptibility to diet-induced steatohepatitis. *Am. J. Pathol.* 176 744–753. 10.2353/ajpath.2010.090667 20019186 PMC2808081

[B4] Chaiyachati B. H. Jani A. Wan Y. Huang H. Flavell R. Chi T. (2013). BRG1-mediated immune tolerance: facilitation of Treg activation and partial independence of chromatin remodelling. *EMBO J.* 32 395–408. 10.1038/emboj.2012.350 23321680 PMC3567501

[B5] Chen B. Fan Z. Sun L. Chen J. Feng Y. Fan X. (2020a). Epigenetic activation of the small GTPase TCL contributes to colorectal cancer cell migration and invasion. *Oncogenesis* 9:86. 10.1038/s41389-020-00269-9 32999272 PMC7528090

[B6] Chen B. Yuan Y. Sun L. Chen J. Yang M. Yin Y. (2020b). MKL1 mediates TGF-β induced RhoJ transcription to promote breast cancer cell migration and invasion. *Front. Cell Dev. Biol.* 8:832. 10.3389/fcell.2020.00832 32984327 PMC7478007

[B7] Chen B. Zhao Q. Xu T. Yu L. Zhuo L. Yang Y. (2020c). BRG1 activates PR65A transcription to regulate NO bioavailability in vascular endothelial cell. *Front. Cell Dev. Biol.* 8:774 10.3389/fcell.2020.00774 PMC7443572 32903816

[B8] Dong W. Kong M. Zhu Y. Shao Y. Wu D. Lu J. (2020). Activation of TWIST transcription by chromatin remodeling protein BRG1 contributes to liver fibrosis in mice. *Front. Cell Dev. Biol.* 8:340. 10.3389/fcell.2020.00340 32478075 PMC7237740

[B9] Fan Z. Kong M. Li M. Hong W. Fan X. Xu Y. (2020). Brahma related Gene 1 (Brg1) regulates cellular cholesterol synthesis by acting as a co-factor for SREBP2. *Front. Cell Dev. Biol.* 8:259. 10.3389/fcell.2020.00259 32500071 PMC7243037

[B10] Fan Z. Li N. Xu Z. Wu J. Fan X. Xu Y. (2019). An interaction between MKL1, BRG1, and C/EBPbeta mediates palmitate induced CRP transcription in hepatocytes. *Biochim. Biophys. Acta Gene Regul. Mech.* 1862:194412. 10.1016/j.bbagrm.2019.194412 31356989

[B11] Fang F. Chen D. Yu L. Dai X. Yang Y. Tian W. (2013). Proinflammatory stimuli engage Brahma related gene 1 and Brahma in endothelial injury. *Circ. Res.* 113 986–996. 10.1161/CIRCRESAHA.113.301296 23963727 PMC4049295

[B12] Feng D. Dai S. Liu F. Ohtake Y. Zhou Z. Wang H. (2016). Cre-inducible human CD59 mediates rapid cell ablation after intermedilysin administration. *J. Clin. Invest.* 126 2321–2333. 10.1172/JCI84921 27159394 PMC4887171

[B13] Fujiwara K. Yasui S. Yonemitsu Y. Arai M. Kanda T. Nakano M. (2015). Importance of the poor prognosis of severe and fulminant hepatitis in the elderly in an era of a highly aging society: analysis in a Japanese center. *Hepatol. Res.* 45 863–871. 10.1111/hepr.12426 25238570

[B14] Gebuhr T. C. Kovalev G. I. Bultman S. Godfrey V. Su L. Magnuson T. (2003). The role of Brg1, a catalytic subunit of mammalian chromatin-remodeling complexes, in T cell development. *J. Exp. Med.* 198 1937–1949. 10.1084/jem.20030714 14676303 PMC2194157

[B15] Heymann F. Hamesch K. Weiskirchen R. Tacke F. (2015). The concanavalin A model of acute hepatitis in mice. *Lab Anim.* 49(1 Suppl.), 12–20. 10.1177/0023677215572841 25835734

[B16] Hong F. Jaruga B. Kim W. H. Radaeva S. El-Assal O. N. Tian Z. (2002). Opposing roles of STAT1 and STAT3 in T cell-mediated hepatitis: regulation by SOCS. *J. Clin. Invest.* 110 1503–1513. 10.1172/JCI15841 12438448 PMC151811

[B17] Huang M. Wang H. Hu X. Cao X. (2019). lncRNA MALAT1 binds chromatin remodeling subunit BRG1 to epigenetically promote inflammation-related hepatocellular carcinoma progression. *Oncoimmunology* 8:1518628. 10.1080/2162402X.2018.1518628 30546959 PMC6287787

[B18] Ichai P. Samuel D. (2008). Etiology and prognosis of fulminant hepatitis in adults. *Liver Transpl.* 14(Suppl. 2), S67–S79. 10.1002/lt.21612 18825677

[B19] Ikeda A. Aoki N. Kido M. Iwamoto S. Nishiura H. Maruoka R. (2014). Progression of autoimmune hepatitis is mediated by IL-18-producing dendritic cells and hepatic CXCL9 expression in mice. *Hepatology* 60 224–236. 10.1002/hep.27087 24700550

[B20] Ikehata M. Yamada A. Morimura N. Itose M. Suzawa T. Shirota T. (2017). Wnt/beta-catenin signaling activates nephronectin expression in osteoblasts. *Biochem. Biophys. Res. Commun.* 484 231–234. 10.1016/j.bbrc.2017.01.053 28093227

[B21] Inagaki F. F. Tanaka M. Inagaki N. F. Yagai T. Sato Y. Sekiguchi K. (2013). Nephronectin is upregulated in acute and chronic hepatitis and aggravates liver injury by recruiting CD4 positive cells. *Biochem. Biophys. Res. Commun.* 430 751–756. 10.1016/j.bbrc.2012.11.076 23206711

[B22] Jaruga B. Hong F. Kim W. H. Gao B. (2004). IFN-gamma/STAT1 acts as a proinflammatory signal in T cell-mediated hepatitis via induction of multiple chemokines and adhesion molecules: a critical role of IRF-1. *Am. J. Physiol. Gastrointest. Liver Physiol.* 287 G1044–G1052. 10.1152/ajpgi.00184.2004 15246962

[B23] Kahai S. Lee S. C. Lee D. Y. Yang J. Li M. Wang C. H. (2009). MicroRNA miR-378 regulates nephronectin expression modulating osteoblast differentiation by targeting GalNT-7. *PLoS One* 4:e7535. 10.1371/journal.pone.0007535 19844573 PMC2760121

[B24] Kong M. Chen X. Lv F. Ren H. Fan Z. Qin H. (2019a). Serum response factor (SRF) promotes ROS generation and hepatic stellate cell activation by epigenetically stimulating NCF1/2 transcription. *Redox Biol.* 26:101302. 10.1016/j.redox.2019.101302 31442911 PMC6831835

[B25] Kong M. Hong W. Shao Y. Lv F. Fan Z. Li P. (2019b). Ablation of serum response factor in hepatic stellate cells attenuates liver fibrosis. *J. Mol. Med.* 97 1521–1533. 10.1007/s00109-019-01831-8 31435710

[B26] Lanthier N. Molendi-Coste O. Cani P. D. van Rooijen N. Horsmans Y. Leclercq I. A. (2011). Kupffer cell depletion prevents but has no therapeutic effect on metabolic and inflammatory changes induced by a high-fat diet. *FASEB J.* 25 4301–4311. 10.1096/fj.11-189472 21873555

[B27] Leifeld L. Dumoulin F. L. Purr I. Janberg K. Trautwein C. Wolff M. (2003). Early up-regulation of chemokine expression in fulminant hepatic failure. *J. Pathol.* 199 335–344. 10.1002/path.1298 12579535

[B28] Li N. Kong M. Zeng S. Hao C. Li M. Li L. (2019a). Brahma related gene 1 (Brg1) contributes to liver regeneration by epigenetically activating the Wnt/beta-catenin pathway in mice. *FASEB J.* 33 327–338. 10.1096/fj.201800197R 30001167

[B29] Li Z. Chen B. Dong W. Kong M. Fan Z. Yu L. (2019b). MKL1 promotes endothelial-to-mesenchymal transition and liver fibrosis by activating TWIST1 transcription. *Cell Death Dis.* 10:899. 10.1038/s41419-019-2101-4 31776330 PMC6881349

[B30] Li Z. Chen B. Dong W. Kong M. Shao Y. Fan Z. (2019c). The chromatin remodeler Brg1 integrates ROS production and endothelial-mesenchymal transition to promote liver fibrosis in mice. *Front. Dev. Cell Biol.* 7:245. 10.3389/fcell.2019.00245 31750301 PMC6842935

[B31] Li Z. Li P. Lu Y. Sun D. Zhang X. Xu Y. (2019d). A non-autonomous role of MKL1 in the activation of hepatic stellate cells. *Biochim. Biophys. Acta Gene Regul. Mech.* 1862 609–618. 10.1016/j.bbagrm.2019.03.001 30951901

[B32] Li Z. Lv F. Dai C. Wang Q. JIang C. Fang M. (2019e). Activation of galectin-3 (LGALS3) transcription by injurious stimuli in the liver is commonly mediated by BRG1. *Front. Cell Dev. Biol.* 7:310. 10.3389/fcell.2019.00310 31850346 PMC6901944

[B33] Li Z. Xia J. Fang M. Xu Y. (2019f). Epigenetic regulation of lung cancer cell proliferation and migration by the chromatin remodeling protein BRG1. *Oncogenesis* 8:66. 10.1038/s41389-019-0174-7 31695026 PMC6834663

[B34] Li N. Liu S. Zhang Y. Yu L. Hu Y. Wu T. (2020a). Transcriptional activation of matricellular protein Spondin2 (SPON2) by BRG1 in vascular endothelial cells promotes macrophage chemotaxis. *Front. Cell Dev. Biol.* 8:794. 10.3389/fcell.2020.00794 32974343 PMC7461951

[B35] Li Z. Kong X. Zhang Y. Yu L. Guo J. Xu Y. (2020b). Dual roles of chromatin remodeling protein BRG1 in angiotensin II-induced endothelial-mesenchymal transition. *Cell Death Dis.* 11:549 10.1038/s41419-020-02744-y PMC7368857 32683412

[B36] Li Z. Zhang Y. Yu L. Xiao B. Li T. Kong X. (2020c). BRG1 stimulates endothelial derived alarmin MRP8 to promote macrophage infiltration in an animal model of cardiac hypertrophy. *Front. Cell Dev. Biol.* 8:569 10.3389/fcell.2020.00569 PMC7358314 32733885

[B37] Liu L. Mao L. Wu X. Wu T. Liu W. Yang Y. (2019a). BRG1 regulates endothelial-derived IL-33 to promote ischemia-reperfusion induced renal injury and fibrosis in mice. *Biochim. Biophys. Acta Mol. Basis Dis.* 1865 2551–2561. 10.1016/j.bbadis.2019.06.015 31228616

[B38] Liu L. Mao L. Xu Y. Wu X. (2019b). Endothelial-specific deletion of Brahma-related gene 1 (BRG1) assuages unilateral ureteral obstruction induced renal injury in mice. *Biochem. Biophys. Res. Commun.* 517 244–252. 10.1016/j.bbrc.2019.07.077 31349970

[B39] Lu Y. Lv F. Kong M. Chen X. Duan Y. Sun D. (2019). A cAbl-MRTF-A feedback loop contributes to hepatic stellate cell activation. *Front. Cell Dev. Biol.* 7:243. 10.3389/fcell.2019.00243 31681772 PMC6805704

[B40] Lv F. Li N. Kong M. Wu J. Fan Z. Miao D. (2020). CDKN2a/p16 antagonizes hepatic stellate cell activation and liver fibrosis by modulating ROS levels. *Front. Cell Dev Biol.* 8:176. 10.3389/fcell.2020.00176 32266258 PMC7105638

[B41] Mao L. Liu L. Zhang T. Qin H. Wu X. Xu Y. (2020a). Histone deacetylase 11 contributes to renal fibrosis by repressing KLF15 transcription. *Front. Cell Dev. Biol.* 8:235. 10.3389/fcell.2020.00235 32363192 PMC7180197

[B42] Mao L. Liu L. Zhang T. Wu X. Xu Y. (2020b). MKL1 mediates TGF-beta-induced CTGF transcription to promote renal fibrosis. *J. Cell Physiol.* 235 4790–4803. 10.1002/jcp.29356 31637729

[B43] Mizuhara H. O’Neill E. Seki N. Ogawa T. Kusunoki C. Otsuka K. (1994). T cell activation-associated hepatic injury: mediation by tumor necrosis factors and protection by interleukin 6. *J. Exp. Med.* 179 1529–1537. 10.1084/jem.179.5.1529 8163936 PMC2191474

[B44] Muller-Deile J. Dannenberg J. Schroder P. Lin M. H. Miner J. H. Chen R. (2017). Podocytes regulate the glomerular basement membrane protein nephronectin by means of miR-378a-3p in glomerular diseases. *Kidney Int.* 92 836–849. 10.1016/j.kint.2017.03.005 28476557 PMC5658661

[B45] Ndekwe P. Ghabril M. S. Zang Y. Mann S. A. Cummings O. W. Lin J. (2017). Substantial hepatic necrosis is prognostic in fulminant liver failure. *World J. Gastroenterol.* 23 4303–4310. 10.3748/wjg.v23.i23.4303 28694671 PMC5483505

[B46] Ohta A. Sekimoto M. Sato M. Koda T. Nishimura S. Iwakura Y. (2000). Indispensable role for TNF-alpha and IFN-gamma at the effector phase of liver injury mediated by Th1 cells specific to hepatitis B virus surface antigen. *J. Immunol.* 165 956–961. 10.4049/jimmunol.165.2.956 10878371

[B47] Okamoto T. Yoshida S. Kobayashi T. Okabe S. (2001). Inhibition of concanavalin A-induced mice hepatitis by coumarin derivatives. *Jpn. J. Pharmacol.* 85 95–97. 10.1254/jjp.85.95 11243581

[B48] Ottoson N. C. Pribila J. T. Chan A. S. Shimizu Y. (2001). Cutting edge: T cell migration regulated by CXCR4 chemokine receptor signaling to ZAP-70 tyrosine kinase. *J. Immunol.* 167 1857–1861. 10.4049/jimmunol.167.4.1857 11489961

[B49] Saiman Y. Friedman S. L. (2012). The role of chemokines in acute liver injury. *Front. Physiol.* 3:213. 10.3389/fphys.2012.00213 22723782 PMC3379724

[B50] Shao J. Weng X. Zhuo L. Yu L. Li Z. Shen K. (2019). Angiotensin II induced CSF1 transcription is mediated by a crosstalk between different epigenetic factors in vascular endothelial cells. *Biochim. Biophys. Acta Gene Regul. Mech.* 1862 1–11. 10.1016/j.bbagrm.2018.10.001 30317027

[B51] Smale S. T. Tarakhovsky A. Natoli G. (2014). Chromatin contributions to the regulation of innate immunity. *Annu. Rev. Immunol.* 32 489–511. 10.1146/annurev-immunol-031210-101303 24555473

[B52] Steigedal T. S. Toraskar J. Redvers R. P. Valla M. Magnussen S. N. Bofin A. M. (2018). Nephronectin is correlated with poor prognosis in breast cancer and promotes metastasis via its integrin-binding motifs. *Neoplasia* 20 387–400. 10.1016/j.neo.2018.02.008 29539586 PMC5909680

[B53] Sun L. Chen B. Wu J. Jiang C. Fan Z. Feng Y. (2020). Epigenetic regulation of a disintegrin and metalloproteinase (ADAM) promotes colorectal cancer cell migration and invasion. *Front. Cell Dev. Biol.* 8:581692. 10.3389/fcell.2020.581692 33043016 PMC7517301

[B54] Sun X. Chuang J. C. Kanchwala M. Wu L. Celen C. Li L. (2016). Suppression of the SWI/SNF component Arid1a promotes mammalian regeneration. *Cell Stem Cell* 18 456–466. 10.1016/j.stem.2016.03.001 27044474 PMC4826298

[B55] Tian W. Xu H. Fang F. Chen Q. Xu Y. Shen A. (2013). Brahma-related gene 1 bridges epigenetic regulation of proinflammatory cytokine production to steatohepatitis in mice. *Hepatology* 58 576–588. 10.1002/hep.26207 23281043

[B56] Tiegs G. Hentschel J. Wendel A. (1992). A T cell-dependent experimental liver injury in mice inducible by concanavalin A. *J. Clin. Invest.* 90 196–203. 10.1172/JCI115836 1634608 PMC443081

[B57] Toraskar J. Magnussen S. N. Chawla K. Svineng G. Steigedal T. S. (2018). Nephronectin mediates p38 MAPK-induced cell viability via its integrin-binding enhancer motif. *FEBS Open Bio* 8 1992–2001. 10.1002/2211-5463.12544 30524949 PMC6275265

[B58] Trotter K. W. Archer T. K. (2008). The BRG1 transcriptional coregulator. *Nucl. Recept. Signal.* 6:e004. 10.1621/nrs.06004 18301784 PMC2254329

[B59] Vangamudi B. Paul T. A. Shah P. K. Kost-Alimova M. Nottebaum L. Shi X. (2015). The SMARCA2/4 ATPase domain surpasses the bromodomain as a drug target in SWI/SNF-mutant cancers: insights from cDNA Rescue and PFI-3 inhibitor studies. *Cancer Res.* 75 3865–3878. 10.1158/0008-5472.CAN-14-3798 26139243 PMC4755107

[B60] Wang B. Kaufmann B. Engleitner T. Lu M. Mogler C. Olsavszky V. (2019). Brg1 promotes liver regeneration after partial hepatectomy via regulation of cell cycle. *Sci. Rep.* 9:2320. 10.1038/s41598-019-38568-w 30787318 PMC6382836

[B61] Wang E. Y. Yeh S. H. Tsai T. F. Huang H. P. Jeng Y. M. Lin W. H. (2011). Depletion of beta-catenin from mature hepatocytes of mice promotes expansion of hepatic progenitor cells and tumor development. *Proc. Natl. Acad. Sci. U.S.A.* 108 18384–18389. 10.1073/pnas.1116386108 22042854 PMC3215019

[B62] Wendon J. Cordoba J. Dhawan A. Larsen F. S. Manns M. Samuel D. (2017). EASL Clinical Practical Guidelines on the management of acute (fulminant) liver failure. *J. Hepatol.* 66 1047–1081. 10.1016/j.jhep.2016.12.003 28417882

[B63] Weng X. Zhang Y. Li Z. Yu L. Xu F. Fang M. (2019). Class II transactivator (CIITA) mediates IFN-gamma induced eNOS repression by enlisting SUV39H1. *Biochim. Biophys. Acta Gene Regul. Mech.* 1862 163–172. 10.1016/j.bbagrm.2019.01.005 30716531

[B64] Wong C. K. Wade-Vallance A. K. Luciani D. S. Brindle P. K. Lynn F. C. Gibson W. T. (2018). The p300 and CBP transcriptional coactivators are required for beta-cell and alpha-cell proliferation. *Diabetes* 67 412–422. 10.2337/db17-0237 29217654

[B65] Wu T. Wang H. Xin X. Yang J. Hou Y. Fang M. (2020). An MRTF-A-Sp1-PDE5 Axis Mediates Angiotensin-II-Induced cardiomyocyte hypertrophy. *Front. Cell Dev. Biol.* 8:839. 10.3389/fcell.2020.00839 33015041 PMC7509415

[B66] Yang Y. Li Z. Guo J. Xu Y. (2020a). Deacetylation of MRTF-A by SIRT1 defies senescence induced down-regulation of collagen type I in fibroblast cells. *Biochim. Biophys. Acta Mol. Basis Dis.* 1866:165723 10.1016/j.bbadis.2020.165723 32061777

[B67] Yang Y. Yang G. Yu L. Lin L. Liu L. Fang M. (2020b). An interplay between MRTF-A and the histone acetyltransferase TIP60 mediates hypoxia-reoxygenation induced iNOS transcription in macrophages. *Front. Cell Dev. Biol.* 8:484. 10.3389/fcell.2020.00484 32626711 PMC7315810

[B68] Yang Y. Liu L. Fang M. Bai H. Xu Y. (2019a). The chromatin remodeling protein BRM regulates the transcription of tight junction proteins: implication in breast cancer metastasis. *Biochim. Biophys. Acta Gene Regul. Mech.* 1862 547–556. 10.1016/j.bbagrm.2019.03.002 30946989

[B69] Yang Y. Liu L. Li M. Cheng X. Fang M. Zeng Q. (2019b). The chromatin remodeling protein BRG1 links ELOVL3 trans-activation to prostate cancer metastasis. *Biochim. Biophys. Acta Gene Regul. Mech.* 1862 834–845. 10.1016/j.bbagrm.2019.05.005 31154107

[B70] Yu C. C. Furukawa M. Kobayashi K. Shikishima C. Cha P. C. Sese J. (2012). Genome-wide DNA methylation and gene expression analyses of monozygotic twins discordant for intelligence levels. *PLoS One* 7:e47081. 10.1371/journal.pone.0047081 23082141 PMC3474830

[B71] Zhang X. Liu S. Weng X. Wu T. Yu L. Xu Y. (2018a). Brg1 trans-activates endothelium-derived colony stimulating factor to promote calcium chloride induced abdominal aortic aneurysm in mice. *J. Mol. Cell Cardiol.* 125 6–17. 10.1016/j.yjmcc.2018.10.012 30336142

[B72] Zhang X. Liu S. Weng X. Zeng S. Yu L. Guo J. (2018b). Brg1 deficiency in vascular endothelial cells blocks neutrophil recruitment and ameliorates cardiac ischemia-reperfusion injury in mice. *Int. J. Cardiol.* 269 250–258. 10.1016/j.ijcard.2018.07.105 30049497

[B73] Zhao Q. Yang J. Chen H. Li J. Que L. Zhu G. (2019). Peli1 induction impairs cardiac microvascular endothelium through Hsp90 dissociation from IRE1alpha. *Biochim. Biophys. Acta Mol. Basis Dis.* 1865 2606–2617. 10.1016/j.bbadis.2019.06.017 31260751

