# Supplementary material for: BRG1 Mediates Nephronectin Activation in Hepatocytes to Promote T Lymphocyte Infiltration in ConA-Induced Hepatitis
Source: Front Cell Dev Biol. 2021 Jan 21;8:587502. doi: 10.3389/fcell.2020.587502 (PMC7858674; doi:10.3389/fcell.2020.587502)
Supplement: Supplementary file 1 [file Data_Sheet_1.pdf]

Hong W *et al*: BRG1 mediates nephronectin activation in hepatocytes to promote T lymphocyte infiltration in ConA-induced hepatitis
Online supplementary material

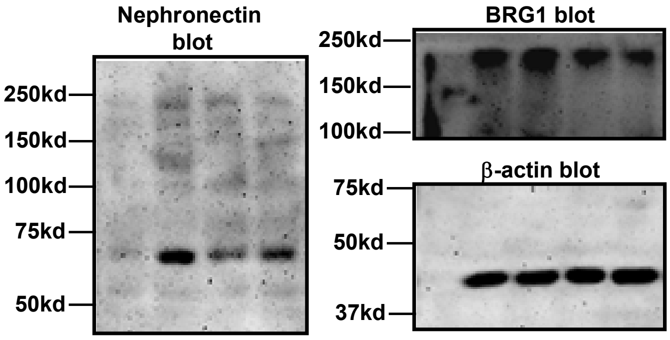

Figure S1: Uncropped full blots for Figure 4B.

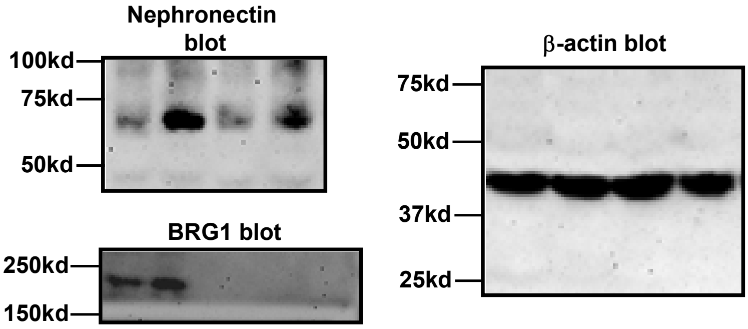

Figure S2: Uncropped full blots for Figure 4F.

Table I: Acute Hepatitis Patient Information Sheet

Patient ID	Gender	Age (yr)	BT (^{o}C)	BP (mmHg)	ALT (U/dL)	AST (U/dL)	LDH (U/dL)	Bilirubin (μM)
1	Female	52	37.8	127/83	243.6	287	618	194.8
2	Male	29	36.5	135/84	231.7	297.2	565	141.6
3	Male	28	36.5	106/71	863.8	2514.2	4627	181.1
4	Female	48	36.2	145/98	245.6	805.7	729	445.3
5	Female	29	36.5	102/59	288.8	215.5	897	196.3
6	Male	45	36.4	138/75	246.5	217.2	329	232.7
7	Male	74	36.6	110/76	935.1	1233.7	1797	252
8	Male	71	36.4	125/78	817.6	564.4	507	363.5
